# Chorioallantoic Membrane Models of Various Avian Species: Differences and Applications

**DOI:** 10.3390/biology10040301

**Published:** 2021-04-06

**Authors:** Barbora Kundeková, Mariana Máčajová, Majlinda Meta, Ivan Čavarga, Boris Bilčík

**Affiliations:** 1 Institute of Animal Biochemistry and Genetics, CBs SAS, 840 05 Bratislava, Slovakia; mariana.macajova@savba.sk (M.M.); majlinda.meta@savba.sk (M.M.); ivan.cavarga@savba.sk (I.Č.); boris.bilcik@savba.sk (B.B.); 2 St. Elizabeth Cancer Institute, 812 50 Bratislava, Slovakia

**Keywords:** chorioallantoic membrane, quail, turkey, duck, 3R

## Abstract

**Simple Summary:**

The chorioallantoic membrane of an avian embryo is a simple, low-cost, low-maintenance, and well-available in vivo animal model with many advantages in the field of scientific experimentation and a multitude of ways of its application. Our review addresses the avian species that are less known as suitable for the chorioallantoic membrane model (CAM) assay than the most commonly used chicken embryo. We describe and compare the characteristics of the quail, turkey, and duck CAM assays, each species offering different advantages for research and opening more possibilities for working methods.

**Abstract:**

The chorioallantoic membrane model (CAM) of an avian embryo is used as an experimental model in various fields of research, including angiogenesis research and drug testing, xenografting and cancer research, and other scientific and commercial disciplines in microbiology, biochemistry, cosmetics, etc. It is a low-cost, low-maintenance, and well-available in vivo animal model that is non-sentient and can be used as an alternative for other mammal experimental models. It respects the principles of the “3R” rule (Replacement, Reduction, and Refinement)—conditions set out for scientific community providing an essential framework for conducting a more human animal research, which is also in line with constantly raising public awareness of welfare and the ethics related to the use of animal experimental models. In this review, we describe the chorioallantoic membrane of an avian embryo, focusing on its properties and development, its advantages and disadvantages as an experimental model, and the possibilities of its application in various fields of biological research. Since the most common chicken CAM model is already well known and described in many publications, we are particularly focusing on the advantages and application of less known avian species that are used for the CAM model—quail, turkey, and duck.

## 1. Introduction

**Figure 1:**
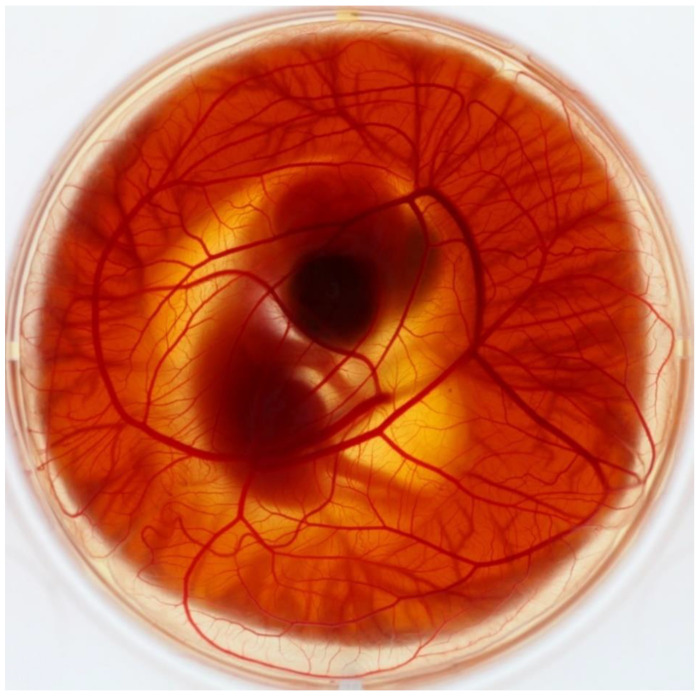
Quail embryo cultivated ex ovo on embryonal day 8 with fully developed chorioallantoic membrane model (CAM).

The chorioallantoic membrane of an avian embryo (CAM) is a simple and rich-vascularized extra-embryonic membrane (Figure 1). During embryonic development, it carries out various functions. Attached to the eggshell, it serves as a breathing organ for the developing embryo, providing gaseous exchange through the pores in the shell [1]. It has a role in osteogenesis during embryonic development, as it provides the calcium supply, removing the mineral from the shell membrane [2]. The CAM actively transports sodium and chloride from the allantoic sac and has a function of an excretory organ for the embryo. It provides a reservoir for waste products such as urea and uric acid [3,4,5,6].

The aim of this review is to summarize the information about the avian embryo chorioallantoic membrane and its application in biological and medical research. Along the well-described chick CAM model, we will also review differences and advantages of other avian species, such as quail, turkey, and duck.

### 1.1. CAM as an Experimental Model

The chorioallantoic membrane model of a developing avian embryo can be used as an in vivo experimental model, which in many countries does not require ethical committee approval [1].

More than fifty years ago, the “3R” rule was declared for more humane research on animal models. William Moy Stratten Russell, together with his colleague Rex Burch, published in 1959 a publication “The Principles of Humane Experimental Technique”, in which they declared three rules to be respected while using laboratory animals. These are replacement, reduction, and refinement. These rules refer to the need for the replacement of the experimental animals for the phylogenetically lowest possible species or forms (including computer models, or in vitro cultures) with reduced ability to feel pain or discomfort. The reduction means lowering their numbers to the necessary minimum and the refinement stands for protecting them from unnecessary suffering [7]. The avian chorioallantoic membrane (CAM) fulfils and respects all of these rules [8].

The first use of this model dates back to 1911, when Rous and Murphy demonstrated the growth of chicken sarcoma tumours transplanted onto the CAM [9]. In the 1930s, the CAM was first used for the cultivation of viruses and bacteria [10,11] and is still used today for the study of vascular development and angiogenesis and general testing of new drugs. It is successfully used in cancer biology, where we can follow all steps of tumour progression: tumour growth, angiogenesis, invasion, extravasation, and metastasis. It is used to study respiratory properties and ion transport in embryogenesis and to test new biomaterials [4]. This system is also used for selective antivascular diagnosis and therapies, like photodynamic therapy, laser photoangiolysis, and radiotherapy [1]. CAM is a useful tool to study short-term transplantation of cryopreserved tissues, for example cryopreserved human ovarian tissues protected before aggressive chemo- or radiotherapy [12].

### 1.2. Advantages and Limitations of the CAM Model

Frequent use of this experimental model undoubtedly has many advantages, such as cost-effectiveness, easy handling, good access to the tissue, and rapid developmental growth, which occurs within a few days [1,6].

The rapid growth and differentiation of the tissue can also represent a disadvantage, as the morphology of the tissue and vascular vessels underlie changes from day to day in the short developmental period. These changes have to be accounted for during the interpretation of the results in which the CAM is in some way experimentally affected. It also allows only a short post-treatment observation time.

The CAM has a rich vascular network well visible through the transparent tissue. The dense vascular vessels and capillaries create suitable conditions for the survival and development of cell and tissue grafts on its surface [13].

The model allows real-time visualization of the assays and complete accessibility to the circulatory system for the intravascular application of substances [1].

The CAM model is naturally immunodeficient. Because of this characteristic, it is possible to transplant cells and tissues from the same or different animal species on its surface, without activating acute immune response [13].

The immunity system of the embryo is slowly developing during embryonic growth. In the case of the chick embryo, the first fully developed lymphocytopoietic organ is the thymus. Lymphoid precursor cells start to appear in the thymus on embryonal day 8 (ED8), followed by the bursa of Fabricius where the lymphoid precursor cells may be recognizable on ED11, and they are more differentiated than the lymphoid cells in the thymus [14,15].

The leukocytes can be detected in the thymus, bursa, or gut from ED10, but a few cells can be found in the yolk sac or spleen already on ED6–7, respectively. The mononuclear phagocytes can be detected in the yolk sac and spleen by ED9, and by ED12, they may also be found in the bursa, gut, and liver [16].

On ED11, T cells can be recognized in the thymus and B cells can be recognized in the bursa of Fabricius by ED12 [16]. Cell-mediated immunity appears by ED13–14 [17].

After ED15, there is a possibility of inducing a nonspecific inflammatory reaction by xenotransplantations, which may have an impact on the success of the grafting and evoke angiogenic reactions and changes in the vasculature, independent of the effect of the graft [1,18].

The embryo becomes immunocompetent on ED18, from when it is capable of an innate and adaptive immune response [16].

The immunodeficiency of an experimental model does not always have to mean an advantage. After inoculation of *Candida albicans* onto the chick CAM surface in an amount of 10^5^–10^8^ cells on ED7–ED10, Gow et al. observed that death of the embryos occurred within 12–24 h. Longer than 24 h after inoculation survived only the embryos that were inoculated by less than 10^4^ yeast cells [19].

The impact on the death rate of chick embryos caused by the number of yeast cells of *Candida albicans* inoculated on CAM was described by Jacobsen et al., who found out that the application of 10^7^ cells led to 75–100% mortality rate, while the application of 10^4^ cells did not have a significant effect on embryo survival. The authors observed a correlation between the embryo survival and the developmental day on which the infection was inflicted. Both concentrations of cells caused a significantly higher mortality rate while applied earlier during the development (on ED8 rather than on ED10). With increasing developmental day, the embryos showed a more balanced immunity response, lowering the production of proinflammatory cytokines and increasing the production of regulatory cytokines. The authors described a protective effect of interleukin 10, which is the proinflammatory response of the embryo to the infection, lowering the inflammation-related damage of the tissue [20].

In most countries, working with chick CAM model does not require an ethical committee approval for animal experimentation, since it was established by The Institutional Animal Care and Use Committee (IACUC), an Association of New England Medical Centre and Tufts, as well as the National Institutes of Health, USA [21], that a chick embryo does not experience pain until ED14 [6,8,22] and until ED17, it is not considered as a living animal [6,23]. In the United Kingdom, the experiments involving the avian embryos that are performed only during the first two-thirds of the incubation period (until ED14) while the embryos are not allowed to survive until the start of the third incubation period, are not regulated by ASPA (Animals (Scientific Procedures) Act 1986 [24]) and do not require a license [25]. The CAM tissue itself is not innervated, and experiments are usually terminated before the development of centres in the brain associated with pain perception [6].

The main advantages of the avian CAM model in comparison with mammal models are lower cost, simplicity of the model, and rapidity of the engraftment. In immunodeficient NOD/SCID mice, for example, it can mean a difference of 14 days for the engraftment in mice and 2 days for detectable engraftment in the case of a turkey embryo. The lower expenses are not only related to the model itself but also to the breeding and maintenance that is required by the animals with suppressed immunity. There are also lower space requirements and it is not necessary to use anaesthetics during the work, as this model does not experience pain [22,26].

One of the limitations and disadvantages of CAM as an experimental model is its avian origin, which implicates differences with mammalian physiology and their drug metabolism. This means that there is also limited availability of reagents, such as antibodies, cytokines, or primers, that are compatible with used avian species [1,6]. The significant differences are also in the physiological requirements and functions of the vasculature [27].

The use of CAM model and the interpretation of results in angiogenesis research, however, may need caution. Its rapid growth and markedly changing and expanding vasculature might obscure any proangiogenic effect of the tested substance, or if the agent would cause local edema, it might indicate a false antiangiogenic effect. The change of density of the vascular network can easily be mistaken with vasodilation or vasoconstriction, by the appearance of the previously invisible small capillaries, or vice versa [27] or due to rearrangement of existing vessels. The angiogenesis observed in the first 24 h can also be easily mistaken with only vasodilatation [6].

The false angiogenic results may be caused by any irritation of the CAM surface inflicted, e.g., by shell dust or sliver produced during egg opening and manipulation [1,27].

## 2. CAM Development and Morphology

As the most commonly used species for the CAM model is chicken (*Gallus gallus*), in this chapter, we are going to describe first the general information about CAM development and morphology, with reference to the chicken embryo, and then we will discuss also the differences in CAM development of other avian species.

In the developing egg of an avian embryo, there are four extraembryonic membranes: the yolk sac, the amnion, the serosa, and the allantois [6].

Histologically, the avian CAM consists of two epithels, the upper chorion attached to the shell membrane and the lower allantois. Between them lies a mesodermal layer of stroma. The allantois, which is of endodermal origin, appears at the ED3 of chick embryonic development as a small, thick-walled pocket growing from the ventral wall of the hindgut. The allantoic vesicle then grows in a period between ED4 and ED10, during which it fuses with the chorion (of ectodermal origin), connecting the two mesodermal layers and forming the chorioallantoic membrane [1,28].

The mesoderm between the allantois and the chorion is composed of a loose matrix of mesenchymal cells and collagen fibrils with a rich vascular and lymphatic network, which is connected with the embryo by two allantoic arteries and one vein, located in the allantoic vesicle [3,6]. During the development, the vascular vessels and capillaries get from the mesenchymal layer to the chorion. As the chorion grows, the capillaries remain at the external part of the layer, keeping proximity with the ambient air [29].

The attachment of the CAM to the shell inner membrane occurs already during the ED4–5 [1]. Before the ED6, the gas exchange of the embryo occurs through the vascularized part of the yolk sac membrane [28].

The growth of CAM is completed on the ED10; by the ED12, it completely covers the inner shell surface, and on the ED13 is the CAM tissue fully differentiated [1,28,29].

Makanya et al. divided the CAM growth of the chicken embryo into three phases: the first phase lasts from ED4–ED5 to ED12 and can be characterized as the phase of growth and proliferation, during which the tripartite structure of the CAM is formed. The second phase, differentiation and expansion, takes from ED13 until ED18. During this phase, the two epithelial layers get thicker and more complex, with more differentiated and specialized cells in the tissue. The last phase is a phase of regression and degeneration of the CAM tissue. It takes from the ED18 until the hatching day, as during this time, the lungs of the embryo start taking over the breathing function and the blood is diverted from the CAM tissue to the pulmonary circulation [29].

### 2.1. Differences in the Embryo Development between Various Avian Species

For a CAM experimental model, there is a possibility to use embryos of various avian species that may have different properties and offer different advantages for the research. One of the main differences between the species is the length of embryonal development, which can last, for example, from 16 days in the case of a quail to 28 days in the case of a turkey embryo.

Ainsworth et al. described the growth and developmental stages of Japanese quail embryo (*Coturnix japonica*), comparing it with the stages of developing chick embryos by Hamburger and Hamilton [30]. They reported that in the beginning, during the first 5.5 days of incubation, the developmental stages of both species are identical. During the middle of the development, the individual stages of quail ontogeny were still morphologically comparable to the chicken, but they were shorter, as the quail ontogeny rate increased. This lasted until the quail ED8–8.5, which corresponded to the chick ED8–9. From this point in the later stages, the morphology could no longer be comparable between them, and the rate of the quail development became even more rapid [31].

Larger species like a turkey or a duck have a slower embryonal development than the chicken embryo. Grinberg et al. compared chick and turkey embryos as a model for tumour cell engraftment and as optimal found ED10 for chick embryos and ED12 for turkey [26]. Capua et al. used for virus inoculation various avian species of different embryonic age, to match a similar developing stage: chick embryo on ED9, turkey embryo on ED10, Muscovy duck on ED11, and mallard duck on ED14 [32], while Annas et al. stated that ED18 of eider duck embryo approximately corresponds to the ED15 of chicken [33].

Lusimbo et al. studied the chorioallantoic membrane development of mallard duck (*Anas platyrhynchos*) between ED12 and ED24 and compared it to the chick CAM development. The length of the duck egg incubation is 26–28 days, which is longer than the 21-days-long development of the chicken embryo. The authors say that the histologic and ultrastructural features of duck during the observed period were very similar to those of a chicken between ED8 and ED20. On ED13, the CAM, loosely adhering to the shell, covered more than 75% of the shell inner surface. On ED14, the CAM’s attachment became firm and from ED16, it covered the entire eggshell surface (which happens on ED12 of chicken development). The chorionic epithelium became fully differentiated on ED14–16, which is corresponding to the chick stage of development on ED10–12 [3].

### 2.2. Vascular System Development

Thanks to its structure and properties, the CAM is a convenient model for angiogenesis research. The tissue is thin and transparent, which offers easy access to the vascular network and allows its easy monitoring.

The angiogenesis of avian embryo’s CAM is influenced by hydrostatic blood pressure, the extracellular matrix and degradation of type IV collagen [3,34,35], and various cytokines, including vascular endothelial growth factor (VEGF), transforming growth factor b, fibroblast growth factor b, and tumour necrosis factor α (TNFα) [36,37,38,39].

The process of embryonal angiogenesis was studied by analysing the morphological structure of chick CAM vasculature during the embryo development. There are three stages of angiogenesis, characterized by different mechanisms of vascular growth. The early stage occurs between ED5 and ED7, with sprouting as the most prevalent angiogenesis mechanism as the new vessels are spreading into the mesenchymal layer. On ED5, the vascular network has many blind-ended capillaries and in some regions, the vascular vessels are still completely missing. On ED6 is the network more homogeneous, with ubiquitous capillaries, growing even more dense on ED7. During the second stage between ED8 and ED12, the architecture of the vascular network changes, the density of the vessels rises, and the sprouting is replaced by the intussusceptive microvascular growth. The third, last stage is on ED13 and ED14 when the CAM is expanding, and the vasculature reaches its morphological maturity [40].

The two rapid growth phases of CAM vessels are on ED10 and after initiation of endothelial cytodifferentiation at ED14 [41]. The vascular system reaches its final shape on ED18, just before hatching [34].

On ED6–10, the capillaries sprout and form a dense plexus. On ED10–12, the vessels in the mesodermal layer can be distinguished to arterioles, with one or two layers of mesenchymal cells developing an adventitia containing fibroblast-like cells and venules surrounded by the mesenchymal cells, the future muscle cells. The vascular network invades the chorion layer between ED10 and ED14 of chick embryo development [6,42].

Lusimbo et al. compared the differences between mallard duck and chick CAM vascular development: the capillaries of the mallard duck fully incorporate into chorionic epithelium at ED14 (that corresponds to the ED10–12 of chick CAM development). On ED16, the CAM reaches its maximum vascular density in the mesodermal layer and the capillaries get close to the inner shell membrane (which happens at ED13–14 in the case of chick CAM) [3].

## 3. Application

The chorioallantoic membrane of an avian embryo is an experimental model used in the research of angiogenesis [43,44], tumour growth, and cancer research [45,46], development of drug and drug delivery systems [47,48], wound healing and tissue repair [17], or virus cultivation [32,49].

Because of its structure and characteristics, CAM is an applicable model for drug toxicity testing [8]. Hen’s Egg Test (HET) [50] was developed as a rapid, low-cost, and sensitive test, which could provide information about embryotoxic, teratogenic, or immunopathological effects of chemical substances, and their potential to irritate mucous membranes. The HETCAM assay can determine the potential of test materials to cause haemorrhage, lysis, and coagulation of the blood vessels, with high predictability for mild and non-irritating test materials [51]. Its properties are appearing to be sufficient to be used as an alternative to the standard Draize eye irritation test [52] that uses rabbit as a model animal, since the test substances show a similar effect on the membrane as on the eye [51]. Kishore et al. tested the irritation potential of various pesticides comparing the HETCAM assay and the in vivo Draize eye irritation test. The results showed good correlation between the two tests, and the authors highlight the potential of HETCAM to refine or reduce the animal use, especially in the field of ocular testing [53].

CAM could be used as an alternative model for a pig buccal mucosa for evaluation of drug absorption, as the CAM tissue is similar to the buccal mucosa in terms of permeation profile and permeability coefficient value. It has the potential to substitute models of pig corneal and retinal epithelium [54].

Concerning animal replacement for research and testing purposes, the model of skin grafts incorporated into the chorioallantoic membrane could be useful for short-term investigations in dermatology [55]. It would make a good alternative for the murine local lymph node assay, used for testing cosmetics and skin allergenicity predictions. It has an advantage in testing directly on human skin instead of models of different mammalian species. The CAM offers more natural physiological conditions for the skin than common cultivation mediums, as it is nourished by the blood of the embryo and observation can last longer [56].

Other than skin or eye irritants, the CAM model could also be used for irritancy testing of vaginal products and medicaments [57].

Irritation of the CAM tissue evokes responses similar to the mammalian models, which makes this model suitable for testing biomaterials and observing the tissue responses for the duration of circa two weeks. It allows the evaluation of acute and chronic inflammatory responses, fibrosis, granulation, and neovascularization of the tissue caused by an injury or implants [4,58,59].

### 3.1. CAM Model Application in Angiogenesis Research

Because of its characteristics and structure, the CAM is a convenient model for angiogenesis research. Its rich capillary network can be used for in vivo testing of pro-angiogenic or anti-angiogenic agents. Unlike other models used for angiogenesis research, the CAM vasculature develops by forming a flat, 2D-like structure within the membrane [37].

The density and changes of the CAM’s capillary network can be evaluated by range of quantitative, or semi-quantitative techniques, such as visual vessel counting, automated approaches, analysing the vessels number, diameter, density, permeability, branch point number, or blood flow [1,44,60,61].

The suitability of quail CAM for angiogenesis research was tested by Parsons-Wingerter et al. using the fractal analysis of the vasculature [62]. They measured the vascular branching pattern by fractal dimension and the vessel density by grid intersection and tested it after application of fibroblast growth factor as an angiogenic stimulator and angiostatin as an angiogenic inhibitor on the CAM vasculature [37]. The third method for CAM’s vascular network morphology analysis was used by Parsons-Wingerter et al., measuring the vessel parameters of all branching generations by the software VESGEN [39,63].

Lubkin et al. reviewed the approaches to measure the CAM’s vasculature and offered a comparison between the use of fractal dimension method and vascular fraction method. They introduced the measuring method, based on dilation and erosion of the image of the vascular tree. They also proposed the three parameters that should distinguish one arterial tree from another and sufficiently characterize the vascular parameters: the vascular fraction, the distance of the vascularized tissue to its vessels (a length), and the flow capacity of the tissue (an area) [64].

The CAM model shows the capability of vascular regeneration comparable to a human eye. The regeneration of CAM vasculature after damage caused by photodynamic therapy led to regrowth of the capillary network in 48 h by sprouting angiogenesis and reperfusion of larger vessels, forming functional neovasculature altered in its morphology and architecture [65].

### 3.2. CAM Model Application in Transplantations and Tumour Grafting Research

The avian CAM model is convenient for transplantations, tumour angiogenesis, and tumour metastasis research for various reasons. It may provide information about tumour properties and behaviour in the in vivo conditions [26], since it offers a natural environment of a developing organism.

The CAM’s rich vascularization forms a good basis for tumour cultivation. It provides the grafted tissue with necessary nutrients, growth factors, and stem cells derived from the other parts of the embryo [6]. With the presence of extracellular matrix proteins like fibronectin, laminin, type I collagen, or integrin α_ν_β_3_, it resembles the physiological environment of tumour cells [66,67,68]. As the CAM is naturally immunodeficient, it accepts cells and tissue fragments from other species without immune reaction [6].

The CAM was successfully used for the study of various types of human carcinoma cell lines, including the glioblastoma [69,70], oesophageal [71], ovarian [72], cervical [73], breast [74], and colorectal [75] tumour cells. The various carcinoma cell lines that were used on the CAM model for photodynamic therapy research were reviewed by Olek et al. [76].

The CAM also appears to be a promising model to study the perineural invasion and for studying various phenotypes of head and neck squamous cell carcinomas and other tumours that surround or invade the nerves [77].

The growth of tumours on the CAM model was divided by Knighton et al. into two phases: the avascular phase, characterized by slow growth during which the host blood vessels do not yet penetrate the tumour, and the second vascular phase starting when the vessels enter the grafted tumour tissue and initiate its rapid growth. They implanted the tumour grafts in the size of 1 mm that were initially grown on rats onto the CAM surface between ED5–ED11. Implanted tumours then exhibited both phases of growth: during the first phase, in the 24 h since the implantation, the original blood vessels in the tumour tissue disappeared, and after 48 h, the central part of the tumour became necrotic. The second phase started after 72 h since the implantation. The new vessels penetrated the tumour, and 96 h after the implantation, small blood vessels grew through the grafted tissue. After 7–8 days since the implantation, the tumours doubled or tripled their size. If the tumour grafts were implanted on the CAM later than on ED12, the tumour tissue did not grow or even diminished. The authors explained it by CAM’s development process when after ED10, the mitotic rate of the CAM endothelium slows down, and the CAM starts to develop its immune system [78].

Hagedorn et al. tested the CAM model’s suitability for tumour transplantation studies. They confirmed that glioblastoma cells U87 grafting led to the growth of tumours with key features of human glioblastoma at cellular and molecular levels occurring in a highly reproducible manner. The glioblastoma tumours grew in an avascular form during the first two days since the application on the CAM, then proceeded to the vascular phase associated with the vascular endothelial growth factor receptor 2-dependent angiogenesis that led to haemorrhage, necrosis, and peritumoral edema [69].

Only the tumour cells with high invasive potential may be capable of crossing the epithelial barrier formed by the chorion and get sustained by the CAM vessels. Hecht et al. inoculated onto quail CAM neuroblastoma cell lines SH-SY5Y and SK-N-AS, transfected with TrkB vectors to increase their invasive potency, and compared their growth on the CAM tissue with the empty vector control cells. The CAMs were inoculated on ED8 and incubated for 7 days. During this time, the control cells produced small, poorly vascularized, 1–2 mm nodules in 4 of 10 eggs, while TrkB-expressing cells formed multiple, well-vascularized, large tumours with diameters up to 15 mm in 9 of 10 eggs [79].

Strojnik et al. compared the growth, histological, and immunohistochemical characteristics of U87 human glioblastoma cells in the mammal model of nude rat brains and avian chick CAM model. Histologically, cytological features of human glioblastomas (like astrocytes, small anaplastic cells, spindle cells, and giant cells) could be observed in both cases. In the rat model, the cell suspension was applied into the brain and the tumours grew sharply demarcated because of the surrounding tissue. In the CAM model, where the cell suspension was applied on surface, the tumour nodules grew in smaller groups, further from the main mass in the place of the application, located in the connective tissue and connected to the vessel walls. Immunohistochemically, the authors labelled the CAM model as sufficiently similar to the rat model and stated that it could make a good alternative for research of tumour invasiveness [70].

Generally, it takes a shorter time for a tumour to grow and metastasize on the CAM than it would on other commonly used animal models. While the tumour growth can take from 3 to 6 weeks on the mammalian model, the tumour xenografts on the CAM surface can become visible and vascularized within 2–5 days after cell inoculation [6].

The disadvantage of a short time period for tumour growth limited by the duration of embryonal development can be solved by the re-engraftments of the implanted cells to another, new CAM [22].

The rapid tumour formation and short time period of the CAM assay are advantageous for the use in anticancer drugs screening of patient-derived tumours. The patient-derived chicken egg model may help to test a variety of drugs grafted to a large number of eggs quickly, inexpensively, and in a live organism, which might allow developing patient-customized drugs and suit the optimal therapy [26,80].

Besides the rapid growth and development of grafted tumour cells and tissue biopsies on the place of implantation, the tumour cells often metastasize to other organs of the embryo. They can grow through the CAM layers, intravasate to the vasculature, migrate through the blood vessels, and invade distant organs, like the liver or the lungs, where they can be easily identifiable. The suitability of the CAM as a model for the study of metastasis migration also supports the longer survival of cancer cells in the CAM microcirculation and higher quantity of successfully extravasated cells in comparison to the rodent models with intravenously applied tumour cells [22,81,82,83].

**Figure 2:**
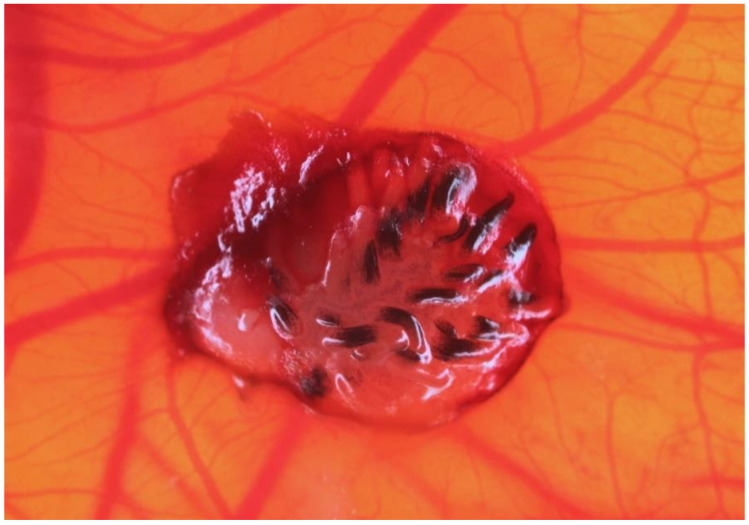
Example of feather bud assay. Quail skin graft placed on CAM surface with developed feather buds, four days after transplantation.

Another type of homologous transplantation is a combined method of CAM model and feather bud (FB) assay [84,85]. It enables to determine the efficiency with which the particular substances induce vascular growth and ensure sufficient blood supply to the target tissue. The richly vascularized chorioallantoic membrane is capable of inducing the spread of its indigenous vasculature even to other, non-related tissues, such as the poorly vascularized avian skin, which, in turn, results in the growth of feathers in that area (Figure 2). The combined CAM/FB method offers several advantages, especially in comparison to other frequently used methods, which are often complicated, time-consuming, and heavily dependent on the provision and supply of exogenous proangiogenic factors. Furthermore, unlike the CAM/FB method, the other methods tend to focus on areas with already developed vasculature, and therefore do not provide any information whatsoever about the ability of tested substances to induce angiogenesis de novo in previously non-vascularized tissues. Chen et al. evaluated the ability of several substances whose proangiogenic properties (fumagilin, minocycline, zoledronic acid, doxorubicin) have already been experimentally proven to induce the development of blood vessels and their subsequent outgrowth, which is accompanied by the growth of feather in previously non-vascularized avian skin. However, after four days after application, the aforementioned substances started showing a concentration-dependent inhibitory effect, especially in comparison to the controls [84].

### 3.3. CAM Model Application in Microbiology and Other Areas

An infected CAM can serve as a convenient model for research of bacteria or yeast virulence, pathogenicity, and invasiveness through the membranes [19,20]. The CAM model was used for the study of invasive potential and interactions of various strains of *Klebsiella pneumoniae*, *Escherichia coli*, or *Salmonella typhimurium* and their combinations. The authors observed the penetration and mortality ratios of the embryos and examined the mode of crossing the epithelial barrier by the bacteria [86].

The response of the chicken embryo to the yeast *Candida albicans* and *Candida glabrata* infection via the CAM was compared to the infected murine model. While in the case of the murine model, frequent occurrence of abscess formation was observed, in the case of the chick CAM model, it was prevailing with the granuloma formation, typical for the avian immune response. There also appeared to be differences in the function of interleukins, where in the contrast to the mammalian model, the IL10 had a beneficial effect in the avian model [20].

The CAM model can also be used to study and test the therapy methods for microbial infections. The CAM with inoculated bacteria can represent an infected tissue for the testing of biomaterials with antimicrobial properties that could be used for patients with chronic wounds [87].

The CAM tissue can reproduce all the phases observed in the wound healing process, such as re-epithelization and hyperplasia of the chorionic epithelium, angiogenesis with three times as many microvessels and fibroblasts in the mesenchyme compared to the unaffected tissue, inflammation with the infiltrate mostly consisting of macrophages, and fibronectin deposition, resulting in scar formation. Therefore, it makes a good in vivo model to study wound repair [17].

The activity and toxicity of newly developed or studied drugs and drug delivery systems can be evaluated by their effect on the CAM and the developing embryo considering the embryo death rate or inflammation and neovascularization of the CAM [47].

It includes the research of photosensitive drugs used for photodynamic diagnosis and therapy. It is a diagnostic and treatment method that uses photoactive compounds, photosensitizers, for their fluorescent activity under the low-wavelength light in the visible spectrum. Using the light of higher intensity, the photosensitizers are capable of destroying the cells of pathological tissue via apoptosis or necrosis [88,89,90]. The characteristics of the CAM model offer many advantages for research in this area including the real-time observation of blood vessels during the treatment and the ability to verify various parameters of photodynamic therapy like type, dose, administration via, incubation interval, light dose, rate of fluency, and irradiation time of the used photosensitizer [47,48]. Olek et al. reviewed the studies that used the CAM model for the research of photodynamic therapy. The CAM was used for the testing of various photosensitive drugs, their pharmacokinetics and light conditions necessary for the photodynamic reaction, and their ability to treat vascular diseases or engrafted carcinomas of various cell lines [76].

The CAM model may also help the research in the field of radiobiology, evaluating the effect of radiation on the CAM tissue, angiogenesis, or growth of engrafted tumours [22].

### 3.4. Quail CAM Model Application

Quail embryo development and angiogenesis in the CAM vascular network were researched even in the conditions of space. Fertilized eggs of Japanese quail (*Coturnix japonica*) were flown on the MIR18 mission as a part of the first joint US–Russian MIR/Shuttle program. The vascular development of the CAM was firstly studied in conditions that simulated the incubation conditions of eggs launched into space on Progress 227 (in terms of vibration and g-force profile) and compared to the vascular development of eggs kept in common laboratory conditions. The simulated conditions affected the normal progress of CAM angiogenesis and caused a decrease in the microvascular density. Affected angiogenesis might have contributed to the survival rate of embryos in space. The eggs that were launched at ED0–2 did not develop, and those that were launched at ED7–10 developed normally, which implies a window during embryonic development that is sensitive to gravitation [91].

#### 3.4.1. Angiogenesis Research

Quail CAM model was used for studying angiogenic effects of vascular endothelial growth factor-165 (VEGF_165_), endothelial nitric oxide synthase (eNOS) [38], transforming growth factor-β1 (TGF-β1) [39], fibroblast growth factor-2 [92], the angiogenic potential of a growth factor mixture derived from bovine bone [93], the anti-inflammatory and antiangiogenic drugs like the steroid triamcinolone acetonide [94], or inhibitory effect of lebein, a heterodimeric disintegrin isolated from *Macrovipera lebetina* snake venom on VEGF-stimulated angiogenesis [95].

Abiuso et al., in their study, tested the ability of histamine receptor H4 agonist 2-(2-guanidinoethyl)isothiourea to negatively affect the pro-angiogenic effect of R2C rat Leydig tumour cell-derived conditioned medium applied on the CAM surface on the film discs [96]. The angiogenic activity of conditioned medium from senescent adipose-derived mesenchymal stem cells on vascularization of the quail CAM was studied by Ratushnyy et al. [97].

Other studies used the quail model to evaluate the pro-angiogenic effect of leptin [98,99], high- and low-molecular heparins [99], nerve growth factor [100], or relasates obtained following platelet-rich plasma coagulation and inhibition of their pro-angiogenic effect by acetylsalicylic acid [2]. It was also used as a preliminary screening method of angiogenic activity and tissue response to biomaterials, such as silicate bioactive glass nanoparticles incorporated into collagen films [47], or porous biopolymer polyhydroxybutyrate/chitosan scaffolds [101].

The quail CAM model was also used for the screening of angiogenic properties of plant derivatives xanthone V1 and 2-acetylfuro-1,4-naphthoquinone, isolated from Cameroonian medicinal plants *Vismia laurentii* and *Newbouldia laevis*, respectively [102], methanol extracts from 34 other spices and plants from Cameroon [103], guieranone A, a naphtyl butanone isolated from the leaves of *Guiera senegalensis* [104], a ligustrazine–betulinic acid derivative [105], tetrahydropalmatine, the index component of *Corydalis yanhusuo* W. T. Wang [106], and extracts of 59 plants used in traditional Korean medicine [107].

Staniszewska et al. used the quail CAM model to study the vascularization of CAM after the application of integrin ligand NoC1, the N-terminal domain of thrombospondin-1. They were interested in its interaction with integrin α9β1 and the involvement of integrin α9β1 in the process of angiogenesis. The pro-angiogenic effect of NoC1 was comparable with the effects of well-known angiogenic growth factors like VEGF or FGF (fibroblast growth factor) and was inhibited by α9β1 inhibitors. These findings observed on the quail CAM model were different from the findings from experiments using the chick CAM model, where the increasing angiogenic response to NoC1 was inhibited by α4β1 integrin antagonists [108].

The quail CAM model was also used for the study of angiogenesis related to ovarian hyperstimulation syndrome (OHSS), which was increased in the number of vascular branch points compared to the control patients. The follicular fluid from patients at risk of OHSS and control patients was applied on the CAMs surface, with the filter-paper discs as carriers. Using this model also analysed the angiogenic effect of different sphingosine-1-phosphate levels in follicular fluid or the effect of inhibition of angiopoietin-1 in follicular fluid on ovarian angiogenesis in both the control and at-risk of OHSS patient groups [109,110].

#### 3.4.2. Xenotransplantations and Angiogenesis Research

The not yet fully functional immune system of the developing embryo allows the engraftment of cells and tissues of different species on the CAM surface. Vasse, in his study, grafted fragments of turtle (*Emys orbicularis*) embryo thymus on the quail CAM and studied their capacity to develop [111]. In another study, the authors implanted onto CAM surface embryoid bodies derived from mouse embryonic stem cells to study the vasculature differentiation process [112].

Brachvogel et al. studied the angiogenic potential of murine perivascular cells by grafting the cell aggregates on quail CAM surface [113].

Besides the angiogenic effect, the lymphangiogenesis was also studied on quail CAM, caused by engraftment of two types of rat tumour cells: 10AS pancreatic carcinoma and C6 glioma cells [114], or VEGF-C expressing human A375 melanoma cells [115].

Interactions between implanted tumour cells and the quail CAM tissue (the microtumours formation, metastatic activity, and capability of penetration into the mesoderm layer) were studied and analysed using implanted chemoresistant ovarian yolk sac tumours of NOY-1 and cisplatin-resistant NOY-1 cell lines [116].

Treatment of CAM implanted tumour cells by various drugs was subject of several studies. Brown et al., on α9β1 integrin positive glioblastoma cell line LN229, applied the nerve growth factor as a ligand and/or the antagonist—the snake venom dimeric disintegrin VLO5, that increased or decreased the tumour growth, respectively [117]. Melanoma cells MV-3 treated by obtustatin (a snake venom KTS-disintegrin) decreased the tumour size and inhibited its neovascularization [118], and C6 glioma cells treated with snake venom vixapatin reduced the C6 induced angiogenic effect [119].

The therapeutic effect of anti-carbonic anhydrase IX antibodies on hypoxic tumours of human squamous cell carcinoma TE-1 cell line engrafted on the quail CAM model was studied by Debreova et al. The application resulted in reduced invasion and extravasation, associated with the process of metastasis formation [120].

Bardet et al. tested on glioblastoma U87-MG tumour xenografts grown on quail CAM the method for cancer treatment using nanosecond pulsed electric fields [121].

Researchers studying angiogenesis and xenotransplantations on the quail CAM model often make use of the existence of quail-specific monoclonal endothelial marker (QH1) [79,101,114].

Gonzáles-Iriarte et al. presented a CAM model of Japanese quail (*Coturnix coturnix japonica*), where they could, by using methods for apoptotic cells labelling with QH1 for endothelial cells localization, estimate the number of apoptotic cells after stimuli of substances with anti-angiogenic properties. This way, the quail CAM assay allowed them to assess if the tested substance is specifically triggering an apoptotic response in endothelial cells, and it could lead to clarification of the mechanisms by which the anti-angiogenic substances affect the vasculature [122]. This method was later used by Cárdenas et al., studying the apoptotic effect of kahweol on endothelial cells and surrounding tissue [123].

#### 3.4.3. Photodynamic Diagnosis and Therapy

In research of photodynamic diagnosis and therapy of cancer, or other non-malignant diseases, the quail CAM model was used in various studies. Buríková et al. used the quail CAM for engraftment of human squamocellular carcinoma TE1 spheroids and their visualization by the photodynamic diagnosis, a fluorescence-based imaging technique, using hypericin as a photosensitizer in combination with low-density lipoprotein as a transport system [71].

The combinations of hypericin with transport systems of low-density lipoprotein and high-density lipoprotein and their suitability for photodynamic therapy and diagnosis was tested also on human breast ductal carcinoma BT 474 and human breast adenocarcinoma SK BR 3 cell lines implanted onto the quail CAM surface, analysing their pharmacokinetics and fluorescence intensity [74].

The suitability of the quail CAM model for tumour cultivation and research of photodynamic therapy was tested by Majernik et al. on colorectal carcinoma HT-29, HCT 116, and CT26.WT cell lines. They analysed the interconnection of microtumours with the tissue and suitability for molecular analysis of gene and protein expression [75].

The quail CAM model was also used for the study of the effect of photodynamic therapy on the tissue and vasculature. In addition to disruption of the CAM tissue itself, changes like capillary haemorrhage and vanishing to thrombosis, lysis, and bleeding of larger vessels, were visible [124].

### 3.5. Turkey CAM Model Application

Model of turkey (*Meleagris gallopavo*) chorioallantoic membrane was used in the study of nanoparticle iron complexes toxicity and uptake by liver and kidneys of the embryo [125], for culturing and research in tumour growth and invasiveness of BeWo choriocarcinoma cell line [126], or human blood malignancies cells and chemotherapy drug testing [26] and for carcinogenicity testing by studying the effect of carcinogen substance diethylnitrosamine on mitochondrial DNA of turkey embryo, where the CAM application method was used for better time and dose control of the chemical influence [127].

Grinberg et al. compared the advantages of using the model of turkey embryo, as a superior host for human blood malignancies, to the characteristics of chick embryo models. In their study, they applied human tumour blood cells into chick and turkey embryos intravenously through the CAM or into the amnion. To make sure that various engraftment results were not caused only by different development time of the two model species, the authors unified the incubation period to 7 days. While in the case of the chick model, the cells of various used laboratory cell lines engrafted only in the CAM and little or none in the bone marrow of the embryo, the use of the turkey embryo model was more resultful, as more cell lines were successfully engrafted, the nodules formed on turkey CAM were larger than in the chicken, and could be detected already after 2 days. Several tested cell lines, including the leukaemia cell lines K562, DAMI, and Jurkat, and lymphoma cell line Raji, were able to form easily detectable masses in turkey CAM. Higher levels of cells were detected also in the bone marrow. The leukaemia cell lines obtained from patients were engrafted only in the turkey embryo model and one third of the cases were successfully engrafted in the embryo bone marrow [26].

### 3.6. Duck CAM Model Application

There are a few species of ducks of which eggs are used for the CAM model: the mallard (*Anas platyrhynchos*), the domestic duck (*Anas platyrhynchos domesticus*), the Philippine duck (*Anas luzonica*), the Muscovy duck (*Cairina moschata*), and the common eider (*Somateria mollissima*).

Among avian species, the mallard duck was considered as a convenient model organism in environmental toxicology. This species represents wild birds better than domesticated chicken, and it is possible to keep it in semi-captive conditions [3].

Duck CAM model was used for testing of angiogenesis effect of quercetin encapsulated in nanoliposomes [128], ethanolic extracts of brown macroalgae species *Turbinaria ornata* and *Padina australis* [129], crude ethanolic extract from *Costus igneus* [130], and ethanolic leaf extract of *Ocimum basilica* [131], crude methanolic leaf extracts of *Ardisia pyramidalis* [132], and crude methanolic extracts of *Quisqualis indica*, *Carmona retusa*, and *Peperomia pellucida* [133], purslane oil from *Portulaca oleracea* L. and its saponifiable and unsaponifiable fractions [134], *Curcuma longa* Linn. tea powder [135], or chloroform extract of shiitake mushroom (*Lentinus edodes* (Berk)) [136].

Besides the effect on the CAM vasculature, many studies were interested in more influencing factors of tested substances. Villaflores et al. studied the anti-angiogenic effect of marine algae *Gracilaria coronopifolia* and its impact on the levels of iron, zinc, and copper [137]. Rodriguez et al. used the duck CAM model to evaluate the anti-inflammatory (using the irritation test modified from Hen’s Egg Test (HET) [50]) and angiogenic activity of encapsulated and non-encapsulated betalains derived from the red dragon fruit [138]. Raga et al. tested the effect of a mixture of bauerenol, α-amyrin, and β-amyrin obtained from air-dried leaves of *Ardisia* cf. *elliptica* on vascular density and damage of CAM and the expression of von Willebrand factor and epithelial membrane antigen [139,140].

Grinberg et al. compared the duck, turkey, and chick CAM model in tumour xenografting. They were tested as hosts for K562 human leukaemia cells that were applied intravascularly through the CAM. The level of engraftment in the duck embryo model was higher than in the case of the chicken model but lower than in the case of the turkey model [26].

Comparison between eider duck CAM and chicken CAM model made Annas et al., studying the metabolic activation of Trp-P-1 and EROD (7-ethoxyresorufin O-deethylase) activity [33].

Some neurobiological studies used duck CAM and its vascular network as a medium for drug application, affecting the developing embryo [141,142].

Virological research used duck CAM for cultivation, histopathology, and DNA isolation of the duck plague virus [49].

Gionti et al. used the duck CAM model for the inoculation of chick cell xenografts infected with Rous sarcoma virus because of immunodeficiency of the model, which allows engraftment of xenogeneic cells and the resistance of duck cells to the used subgroup A virus [143].

Capua et al. used CAMs of various avian species, such as chicken, turkey, Muscovy duck, and mallard for research on H7N1 low pathogenicity avian influenza. Isolates of the virus were inoculated into the allantoic cavity and the CAM, among other tissues of the developing embryos, was examined by immunohistochemistry. In the four used species, the presence of viral antigen was detected for all isolates only in the allantoic epithelium of the CAM, and one of the isolates replicated also in the chorionic epithelium and the vascular endothelium of the CAM [32].

## 4. Methods and Protocols of CAM Assay Application

Embryo development depends on various inner and environmental factors. Even eggs that are put into the incubator at the same time, can after a few days show little differences in the developmental stage. These factors include genetic differences between various breeds, the season, potential differences in the developmental phase before the start of the incubation, the duration and conditions during the storage of the eggs, and their temperature before incubation. The development may be impacted also by the size of the eggs, the temperature setting and changes in temperature during the incubation, and also by the size and type of the incubator [30]. Depending on the source and strain of eggs used, the developmental age may vary up to 12–18 h [61].

The length of the embryonal development determines the experimental window during which it is possible to work with the CAM (Table 1). This period starts on the embryonal day on which the CAM has sufficient size, as very early manipulation may lead to a higher mortality rate of the embryos. It is common to end the experimentation before the embryonal day when the embryo obtains the capability of feeling the pain. Depending on the individual country regulations, using the CAM model after this stage requires ethical committee approval [1,8]. For example, in the case of a turkey embryo, the maximal incubation time is about 2 weeks, as approximately 3 days before hatching, the embryos are thought to be developed enough to feel the pain [26]. The length of the developmental period of Muskovy duck embryo reaches 35–37 days, which could extend the experimental window even more [144].

According to Nowak-Sliwinska et al., in the case of the chick CAM model, the optimal time for engraftments onto the CAM for the purpose of angiogenesis research is ED7. During this time, the immune system is not yet developed, however, the immature mesoderm offers the graft a befitting environment for the growth. The CAM is less vascularized between the big vessels, so the angiogenic changes are easier to observe and evaluate [61].

### 4.1. In Ovo and Ex Ovo Incubation Method

Embryo incubation is possible in ex ovo or in ovo culture conditions. The eggs are first kept in a humidified incubator for three days (chick or quail embryo) [1,100]. The eggshell must be opened or ruptured by ED3 because after this time, the CAM starts growing and there is the risk of rupturing the CAM or damaging the vasculature [1].

During in ovo cultivation, the eggs must be rotated during the first 3 days of the incubation period to prevent embryos from sticking to the shell membranes. A small hole is then made to the top of the shell, consequently covered with a wrapping film. After this, the eggs are left in the incubator, now without rotating, until the start of the experiment. This procedure changes the pressure in the egg and prevents a growing CAM from binding with the shell membrane. When the CAM is grown enough to work with, the hole is extended to allow access to the chorioallantoic membrane [1]. Sealing of the opening in the shell while the egg is in the incubator is critical for the CAM assay, as the membrane is sensitive to changes in oxygen tension [27].

This method of embryo cultivation is more suitable for longer-lasting experiments, since it provides a more physiological environment and the source of calcium for the embryo is kept. It results in fewer developmental abnormalities and the embryos can reach hatching [47,146].

On the other hand, the cut window may cause a limitation of the working area, which may cause difficulties in high-resolution microscopy on embryos in ovo. Additionally, the pieces of eggshell spilled on CAM surface pieces may induce angiogenesis [6,47,146].

Ex ovo cultivation obtains the transfer of the embryo to the Petri dishes, cultivation plates, or other fitting containers on the third day of embryonal development. This shell-less cultivation approach enables more access to the CAM and provides a bigger working area, which leads to easier manipulation and allows continuous, real-time observation and visualization of the experiment [27].

On the other hand, ex ovo cultivation has higher requirements for sterility and the artificial environment and occurrence of the rupture of the yolk during the manipulation have an impact on the embryo’s survival [1,6,47]. Some authors report survival rates above 80% [58], but generally, the ex ovo cultivation may lead to more extensive drying of the embryos, it lowers the viability, represents a greater risk of infection, and causes a higher mortality rate [6,61,146].

Avian species with larger eggs, like chicken, turkey, or duck, are more suitable and more commonly used for in ovo assay [125,137], although some studies use the ex ovo method [133]. On the other hand, species with small eggs, e.g., quail, are predominantly used in ex ovo method with some exceptions [97]. The quail chorioallantoic membrane ex ovo assay was described in detail by Lazarovici et al., as a model to study the effect of nerve growth factor [100].

### 4.2. Substance Application on the CAM

The experimental substances can be applied topically, injected intravascularly, or into the amnion. Compared with the application to the albumen, the CAM application method may provide more information and better definitions of time and dose dependence of the induced effect [127].

The liquid substances applied topically onto the CAM surface need a supporting carrier for keeping the drug within selected area and marking the place of application. These carriers could be in the form of a ring, a foil, or a sponge. They may cause irritation of the CAM or affect the architecture of the developing vasculature, especially when applied before ED10 (in the chick embryo) depending on the time of the application, weight, and form of the carrier.

Dohle et al. reported the least additional irritation of the CAM tissue using a filter paper as a drug carrier [147]. According to our study of the effect of silicone rings on the CAM angiogenesis, there were no significant differences in vascular density and fractal dimension parameters between the control group and groups with applied silicone rings of various thickness and weight [148].

### 4.3. Differences in CAM Assays of Various Avian Species

Another frequently used avian species for the CAM model is the Japanese quail (*Coturnix japonica*). Despite many similarities, the quail CAM model may have several advantages in comparison with more common chick’s CAM assay. Japanese quails are easy to breed, reach sexual maturation and start to lay eggs in less than 40 days, and produce a large number of eggs. The development of the embryo is shorter, which can be suitable for some experiments and allows high turnover of experiments [124,149].

**Figure 3:**
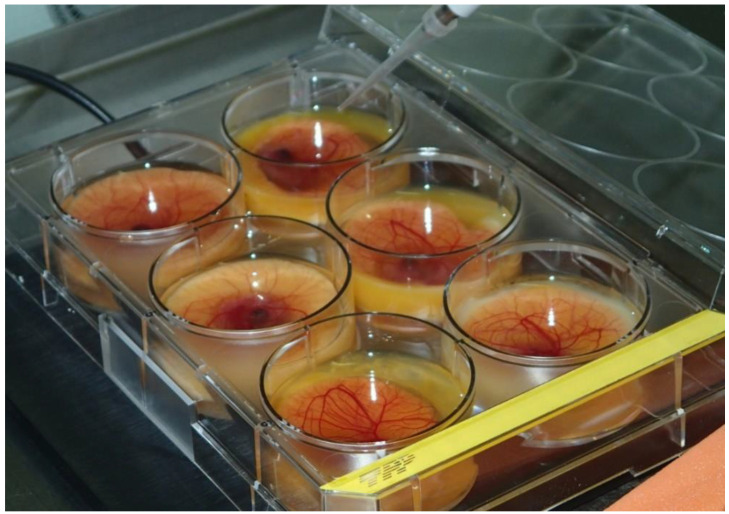
Quail CAM, ex ovo cultivation in 6-well plastic culture plate.

The thinner shell is easier to open, which makes the ex-ovo cultivation and manipulation easier, compared to the chicken embryos [124]. The quail embryos are smaller, which allows them to be incubated in smaller containers, like 6-well cultivation plates, that require less space in the incubator (Figure 3).

Among the disadvantages of using quail for CAM assay is a more fragile constitution of the embryo and mottled pigmentation of Japanese quail eggshells, which make it difficult to stage the eggs by the candling method [124,149].

**Figure 4:**
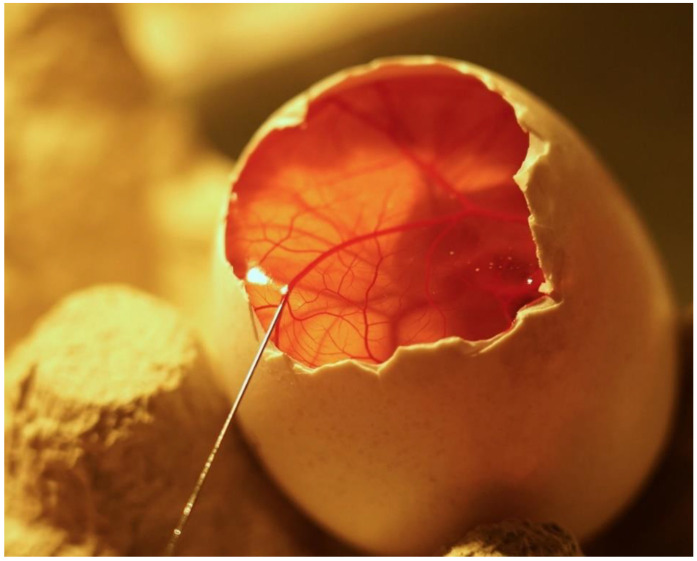
Turkey CAM, in ovo cultivation. Example of intravenous substance application with Hamilton syringe.

It is good to consider the size of the embryo and choose the avian species with the experimental protocol in mind. For example, the vessels of the turkey and chick embryo CAM are more suitable for intravascular application (Figure 4) of substances because of their diameter [81,150], in comparison to the quail model.

Unlike in the chick CAM model, the smaller size of the quail CAM limits the application of multiple experimental substances. On the quail CAM model, Abiuso et al. placed three film discs on one CAM simultaneously [96], while the chick CAM is large enough for application of up to six different samples [124,147].

## 5. Conclusions

Despite the fact, that the chick CAM model is one of the most exploited models in scientific experimental work, the utilization of unconventional avian CAM models such as quail, duck, and turkey may offer more possibilities in experimental work and present some convenient advantages, mostly due to technical reasons. The relative availability of poultry breeding facilities close to experimental laboratories as well as the consequential logistics and underlying costs may be significant in the selection of a model organism. The quail model requires less space for breeding, while providing more eggs in return, which, in addition to the size of the eggs that require less space in the laboratory during the cultivation, allows the utilization of higher number of embryos during the experiment. Furthermore, the different developmental time of other avian species, shorter or longer, may be more suitable for individual experimental designs.

The need for the replacement of the experimental animals for the phylogenetically lowest possible species or forms (including computer models, or in vitro cultures) with reduced ability to feel pain or discomfort is still an acute challenge in many fields of scientifical research. Other requirements involve the reduction, which means lowering the numbers of animals used as model organisms for scientific purposes to the necessary minimum, and the refinement, which stands for their protection from unnecessary suffering, guaranteeing treatment and living in accordance to welfare principles. The avian chorioallantoic membrane assay fulfils and respects all of these rules and appears to be a convenient model for many fields of research.

## Figures and Tables

**Table 1:** The development duration of various avian species embryo and the recommended time period for which they are suitable for experimentation. The development duration is represented by the blue colour, the possible experimental period is represented by the orange colour.

Species	Development Duration								Experimental Period							Experimental Window								References				
*Gallus gallus*	21								ED7–ED14 (* ED18)							8–12 days								[61]				
*Coturnix japonica*	16								ED6–ED12							7 days								[118]				
*Meleagris gallopavo*	28–30								ED10–ED25							14–15 days								[26,126]				
*Anas platyrynchos*	27–28								ED7–ED24							15–18 days								[140,145]				
**Species**	**1**	**2**	**3**	**4**	**5**	**6**	**7**	**8**	**9**	**10**	**11**	**12**	**13**	**14**	**15**	**16**	**17**	**18**	**19**	**20**	**21**	**22**	**23**	**24**	**25**	**26**	**27**	**28**
*Gallus gallus*							12 days																					
*Coturnic japonica*						7 days																						
*Meleagris gallopavo*										16 days																		
*Anas platyrynchos*							18 days																					

* Depending on the country legislative.

## Data Availability

Not applicable.
